# Epistatic effects of Siglec-G and DNase1 or DNase1l3 deficiencies in the development of systemic lupus erythematosus

**DOI:** 10.3389/fimmu.2023.1095830

**Published:** 2023-03-08

**Authors:** Marina A. Korn, Marie Steffensen, Carolin Brandl, Dmytro Royzman, Christoph Daniel, Thomas H. Winkler, Lars Nitschke

**Affiliations:** ^1^ Division of Genetics, Department of Biology, University of Erlangen, Erlangen, Germany; ^2^ Department of Immune Modulation, University Hospital of Erlangen, Erlangen, Germany; ^3^ Department of Nephropathology, University Hospital of Erlangen, Erlangen, Germany

**Keywords:** B cell signaling, mouse models, inhibitory receptors, lymphocytes, autoimmunity

## Abstract

Systemic lupus erythematosus (SLE) is a severe autoimmune disease that displays considerable heterogeneity not only in its symptoms, but also in its environmental and genetic causes. Studies in SLE patients have revealed that many genetic variants contribute to disease development. However, often its etiology remains unknown. Existing efforts to determine this etiology have focused on SLE in mouse models revealing not only that mutations in specific genes lead to SLE development, but also that epistatic effects of several gene mutations significantly amplify disease manifestation. Genome-wide association studies for SLE have identified loci involved in the two biological processes of immune complex clearance and lymphocyte signaling. Deficiency in an inhibitory receptor expressed on B lymphocytes, Siglec-G, has been shown to trigger SLE development in aging mice, as have mutations in DNA degrading DNase1 and DNase1l3, that are involved in clearance of DNA-containing immune complexes. Here, we analyze the development of SLE-like symptoms in mice deficient in either *Siglecg* and *DNase1* or *Siglecg* and *DNase1l3* to evaluate potential epistatic effects of these genes. We found that germinal center B cells and follicular helper T cells were increased in aging *Siglecg* ^-/-^ x *Dnase1* ^-/-^ mice. In contrast, anti-dsDNA antibodies and anti-nuclear antibodies were strongly increased in aging *Siglecg^-/-^* x *Dnase1l3^-/-^* mice, when compared to single-deficient mice. Histological analysis of the kidneys revealed glomerulonephritis in both *Siglecg* ^-/-^ x *Dnase1* ^-/-^ and *Siglecg^-/-^* x *Dnase1l3^-/-^* mice, but with a stronger glomerular damage in the latter. Collectively, these findings underscore the impact of the epistatic effects of *Siglecg* with *DNase1* and *Dnase1l3* on disease manifestation and highlight the potential combinatory effects of other gene mutations in SLE.

## Introduction

Systemic lupus erythematosus (SLE) is a severe autoimmune disease affecting up to 30 of 100,000 individuals worldwide, with a predominance in women of childbearing age (1). As a multi-system disease involving almost all organs, the clinical manifestations of SLE include (but are not limited to) arthritis, cutaneous lupus and lupus nephritis (2). However, the causes for SLE development are not entirely elucidated and appear to comprise environmental as well as multiple genetic factors. A high heritability of SLE was demonstrated by twin studies comprising several families (3, 4). SLE is proposed to be a polygenic disorder despite the discovery of a few monogenic forms of SLE resulting from mutations of, among others, complement factor C1q, factors responsible for apoptosis or DNA clearance, DNA damage repair, sensing of nucleic acids and the production of type I interferon (5), as well as genes associated with the major histocompatibility complex (6) and Fc receptor genes (7). Beside these monogenic causes it is assumed that epistatic effects and accumulation of common single-nucleotide polymorphisms with mild effect in combination with environmental factors result in a predisposition to lupus. Genome-wide association studies in SLE have identified gene variants which are involved in three types of biological processes: immune complex processing; TLR function with type I interferon production; and signal transduction in lymphocytes (8). Additionally, rare frequency genetic variants in signaling proteins were identified by whole genome-sequencing to contribute to the development of SLE (9).

A common mechanism provoking the development of SLE is the break in self-tolerance and thereby the formation of autoimmunity against nuclear components. Self-reactive B cells producing autoantibodies take on a major role in the course of the disease. Autoantibodies directed against double-stranded DNA (anti-dsDNA antibodies) and anti-nuclear antibodies (ANAs) are typical immunological parameters used for the clinical diagnosis of SLE (10). These autoantibodies to nuclear components are formed as SLE is often characterized by defective apoptotic clearance processes (11). Autoantibodies binding to their cognate self-antigen cause the formation of immune complexes, resulting in the production of pro-inflammatory cytokines by immune cells and immune complex deposition in several tissues and organs, thus generating an inflammatory state causing organ injuries (12).

The development of SLE has been studied in mouse models for many years and provided significant insight into mechanisms and possible treatments of SLE. The most well-known mouse models for SLE are the NZB/NZWF1 mice and MRL/lpr mice which spontaneously develop many SLE-like symptoms (13). Additionally, numerous knockout and transgenic mouse models have been generated to investigate the impact of specific genes and gene products on SLE development, for example DNase knockout mice. Mutations of DNases, which are endonucleases degrading DNA, have been found to promote SLE development in both humans and mice. DNases associated with SLE susceptibility belong to the DNase1 family (comprising DNase1, DNase1L1, DNase1L2, DNase1L3) (14). The development of SLE in the absence of DNases was associated with impaired DNA clearance resulting in the formation of DNA complexes in the serum causing the development of autoantibodies (15, 16). DNase1 is mostly expressed by exocrine cells (like lacrimal and salivary glands), the pancreas as well as in kidney, but it is also present in the blood (17). Mutations in *DNASE1* were associated with SLE disease in humans (18). Additionally, *Dnase1* ^-/-^ mice were shown to develop SLE-like disease with increased titers of ANAs and prevalence of glomerulonephritis (16). However, these findings are controversial, as a following analysis of this mouse strain revealed an additional deficiency for tumor necrosis factor receptor-associated protein-1 (Trap1) caused be the deletion of all 9 exons of the *Dnase1* locus (19). Polymorphisms of Trap1 are associated with increased susceptibility to SLE (20). Nevertheless, another *Dnase1* ^-/-^ mouse strain with intact *Trap1* was later reported to develop mild SLE-like disease symptoms (21).

Deficiencies in *DNASE1L3* that were associated with SLE disease have been identified in several human families (22, 23). DNase1l3 is mainly secreted by dendritic cells and macrophages from intestine, spleen and liver; additionally, marginal zone B cells as well as B1a cells show low *Dnase1l3* expression in mice (15, 22). *Dnase1l3^-/-^* mice were shown to develop a SLE-like disease characterized by increased levels of autoantibodies directed against dsDNA and chromatin starting at the age of five weeks. They also developed splenomegaly and glomerulonephritis, as well as spontaneous formation of germinal centers and increased numbers of germinal center B cells (GC B cells) and activated T cells (15). The latter phenotypes were dependent on the genetic background, however (15, 22). Emphasizing the importance of epistatic effects in the development of SLE, the combination of a *Fcgr2b* deficiency and a *Dnase1l3* deficiency was shown to result in enhanced SLE-like disease in comparison to the individual knockouts, as for example the serum levels of dsDNA autoantibodies increased by a factor of 40 (22).

Another important mechanism to maintain self-tolerance is the expression of inhibitory receptors on B cells. Mutations in inhibitory receptors such as the FcγRIIb or the Siglecs CD22 or Siglec-G, as well as in downstream inhibitory signaling proteins such as Lyn or SHP-1 lead to SLE-like disease in mice (24–28). Siglec-G is a member of the Sialic acid-binding immunoglobulin-like lectin (Siglec) family. Siglecs are transmembrane proteins which bind with their extracellular domain to sialic acid containing glycoconjugates and usually have intracellular immunoreceptor tyrosine-based inhibitory motifs (ITIM) that allow them to recruit inhibitory phosphatases such as SHP-1 (29, 30). *Siglecg* deficiency was shown to cause autoimmunity with high titers of autoantibodies and mild glomerular kidney damage in aging mice (31). *Siglecg* deficiency also increases severity of collagen-induced arthritis and SLE-like disease in MRL/lpr mice (32). Despite the involvement of Siglec-G in prevention of the development of SLE-like disease in mice, no association of genetic polymorphisms or mutations of the human orthologue to *Siglecg*, *SIGLEC10* has been detected so far. However, *SIGLEC6* (33) and *SIGLEC12* (34) have been identified as risk loci for SLE development.

Here we study the epistatic effects of genes involved in clearance of immune complexes and inhibitory signaling processes. We investigated combinatory effects of *Dnase* deficiencies and a *Siglecg* deficiency in mice. All single gene-deficient mice (*Dnase1* ^-/-^, *Dnase1l3* ^-/-^ and *Siglecg* ^-/-^ mice) were previously shown to have increased susceptibility to SLE-like disease to some extent (15, 21, 31). Here, we provide evidence for an enhanced susceptibility for SLE in aging *Dnase1l3* x *Siglecg* double-deficient mice with increased titers of anti-dsDNA antibodies as well as ANAs, and elevated nephritis in comparison to single knockout mice. In contrast, *Dnase1* x *Siglecg* double-deficient did only show increased numbers of germinal center cells, but no clear elevation of autoantibodies.

## Materials and methods

### Mice

*Dnase1l3^-/-^* mice (22) were generated as described. *Siglecg* ^-/-^ mice were generated as described (35) and were backcrossed to the C57BL/6 background by marker-assisted speed congenics (31). *Dnase1* mutant mice were generated by CRISPR/Cas9 mediated mutagenesis in JM8A3 embryonic stem (ES) cells from C57BL/6N origin (36). In brief, ES cells were transfected with pX458 (obtained from Addgene, Watertown, MA, (37)) in which the *Dnase1* specific gRNA-sequence 5´ TGACATCGCTGTTATCCAAG 3´ was inserted. GFP-expressing ES cells were sorted and mutations in individual ES cell clones were analyzed by sequencing the amplicon generated by primers flanking the target sequence in exon 3. One clone showing a 65 bp deletion from intron 2 into exon 3 was selected for blastocyst injections, generation of chimeric mice and further breeding with C57BL/6N mice. The deletion does not allow splicing into exon 3, and potential alternative splicing into exons 4, 5 or 6 containing the active sites for Dnase1 enzymatic activity lead to frameshift mutations and premature stop codons. For genotyping of mice these primers were used: DNAase1forw: 5´GGGAGGGACAAAGTCTGAGGTC3´ and DNAase1rev 5´AACAATAGAGCACAGAGGGCGT 3´ creating a 230 bp wildtype product and a 165 bp band for the mutant allele.

### Detection of anti-nuclear antibodies in the serum

Anti-nuclear antibodies in the sera of aging mice were detected using Hep-2 cell slides (Bio-Rad). For this purpose, serum was diluted 1:250 in 1x PBS (Gibco) containing 0.1% BSA (Roth) and 0.05% sodium azide (Sigma-Aldrich). Buffer without serum was used as a negative control, and sera from sick MRL/lpr mice were carried as positive controls at the same dilution. 50 µl of the dilutions were pipetted as drops onto the wells of the slides and incubated for 30 minutes at room temperature in a humid chamber. The drops were then discarded and the slides were washed with 1x PBS for 10 min. Detection of anti-nuclear antibodies was performed with an Fc-specific goat-anti-mouse IgG antibody labeled in AF488 (Jackson ImmunoResearch). 50 µl of the diluted goat-anti-mouse antibody (1:500 in 1x PBS containing 0.1% BSA and 0.05% sodium azide) was pipetted onto the wells and incubated for 30 minutes at room temperature in a dark and humid chamber. Again, the slides were washed for 10 minutes in 1x PBS. After that, the slides were covered with cover glasses (Paul Marienfeld) using DePeX mounting medium (Serva). Data were acquired on an Axio Scope.A1 fluorescence microscope (Zeiss) with identical exposure times for all samples. The images were analyzed using ImageJ software by determining the average pixel intensity of ten nuclei per sample.

### Detection of anti- dsDNA antibodies in the serum

Anti-double stranded DNA antibodies in the serum of aging mice were detected using an ELISA-based method. For this purpose, the wells of a maxisorp plate (96-Well plate; NuncImmuno) were coated with 20 µg/ml Poly-L-Lysine (Sigma-Aldrich) in TE buffer (10 mM Tris and 1 mM EDTA in H_2_O, pH 8). 50 µl per well was used and incubation was performed for two hours at room temperature or overnight at 4°C. The plates were then washed three times in TE buffer before 100 µl of 20 µg/ml calf thymus DNA (Sigma-Aldrich) in TE buffer was added to the wells. This second coating step was carried out overnight at 4°C and afterwards the plates were washed three times with 1xPBS/0.05%Tween20. Wells were then blocked with 150 µl of ELISA buffer (1x PBS containing 0,05% Tween20, 2% FCS, pH 7,4) for one hour at room temperature. The blocking solution was discarded and 50 µl of the serum dilutions (1:100 in ELISA buffer) were pipetted into the wells in duplicates. Pooled serum from NZB/NZW F1 mice was used as standard and ELISA buffer without serum was used as blank. The serum dilutions were incubated for one hour at room temperature. Afterwards, the plates were again washed three times with 1x PBS/0.05%Tween20. Detection of anti-dsDNA-antibodies was performed by incubation with an Fc-specific goat anti-mouse IgG-HRP (Jackson ImmunoResearch) secondary antibody (50 µl, 1:5000 diluted in ELISA buffer) for one hour at room temperature. The plates were washed three times before 50 µl Substrate (o-Phenylenediamine dihydrochloride tablet (Sigma-Aldrich) to a final concentration of 1 mg/ml in 20 mM Na_2_HPO_4_ and 10 mM C_6_H_8_O_7_ and 3% H_2_O_2_) was added into the wells. Before saturation was reached, 50 µl of Stop solution was added (4M H_2_SO_4_) and the optical density was measured at 492-620nm wavelength using the Plate reader and SoftMax Pro software. The amount of anti-dsDNA antibody was determined as a relative unit (arbitrary unit) compared with the pooled serum of NZB/NZW F1 mice.

### Measurement of the blood urea nitrogen content in the serum

For the measurements of the blood urea nitrogen (BUN) content in the sera of aging mice the TECO-Diagnostics BUN kit was used. According to manufacturer`s protocol, 150 µl of enzyme reagent was pipetted in the wells of a 96 well MaxiSorp plate (Nunc-Immuno) and 1 µl Serum was added. 1 µl of pooled serum from sick MRL/Lpr mice was used as a positive control and for negative reference no serum was added. The standard included in the kit had a concentration of 20 mg/dl and was also diluted 1:150 in enzyme reagent and was used in triplicates. The samples were incubated for 10 minutes at room temperature. Following this, 100 µl color reagent were added and followed by an incubation step of 10 minutes at room temperature (dark). Optical density was measured in the ELISA reader at 600 nm wavelength using SoftMax Pro software. The following formula was used to determine the urea nitrogen content in the blood: $BUN\;\left(\frac{mg}{dl}\right)=\frac{oD\;sample}{oD\;standard}\times 20mg/dl$ .

### Cell preparation and flow cytometry analysis

Mice at 42-48 weeks of age or 60 weeks of age were sacrificed and the analyzed organs were collected. For the analysis of bone marrow cells, cells were isolated from one hind leg (femur and tibia). Single cell suspensions were prepared in 1x PBS (Gibco) using a 70 µm cell strainer (Greiner) and cell suspensions were kept at 4°C. Then the cells were pelleted by centrifugation (1300 rpm, 4°C, 10 minutes). After discarding the supernatant, the cells were taken up in ACK buffer (1,5 M NH_4_Cl, 100 mM NaHCO_3_, 10 mM EDTA in H_2_O) (2 ml for bone marrow cells, 3 ml for splenic cells) for erythrocyte lysis. Red cell lysis was performed precisely for three minutes at room temperature before adding twice the volume of 1x PBS and centrifuging the cells. Cells were then taken up in 1x PBS and were counted using trypan blue staining (Gibco) and the Neubauer counting chamber. 4x10^6^ cells were used per staining. Therefore, the cells were transferred into 5 ml FACS tubes and after centrifugation the cells were taken up in 50 µl of the corresponding antibody mix in FACS buffer (1x PBS containing 0,1% BSA, 2mM EDTA, 2mM sodium azide). Staining was carried out for 30 minutes at 4°C protected from light. Cells were then washed with 1 ml FACS buffer and centrifuged. If the staining mix contained biotinylated antibodies, the cells were taken up in 50 µl FACS buffer containing fluorescent labeled streptavidin and incubated again for 30 minutes at 4°C. After a washing step the cells were then taken up in FACS buffer and were measured on flow cytometer CytoflexS (Beckman Coulter). The raw data were analysed using the FlowJo software.

The following antibodies were used for flow cytometer analyses: anti-PD1 PE (clone RMP1-30, BioLegend), anti-CD8-PE/Cy7 (clone 53-6.7, BioLegend), anti-CD44-APC (clone IM7, eBioscience), anti-CD62L-AF700 (clone MEL-14, BioLegend), anti-CXCR5-FITC (clone L138D7, BioLegend), anti-CD4-BV421 (clone GK1.5, BioLegend), GL7-PE (clone GL7, BioLegend), anti-Fas/CD95-PE/Cy7 (clone Jo2, BD Pharmingen), anti-B220-PerCPCy5.5 (clone RA3-6B2, BioLegend), anti-CD138-bio (clone 281-2, BioLegend), anti-TACI/CD267-APC (clone ebio8F10-3, eBioscience), anti-B220-AF700 (clone RA3-6B2, BioLegend) anti-CD21-bio (clone 7E9, BioLegend), anti-CD23-PE (clone B3B4, eBioscience), anti-CD5-PE (clone 53-7.3, BD Pharmingen).

### Histology analysis

For histological analysis, kidneys were fixed with 4% paraformaldehyde in phosphate buffered saline (PBS), dehydrated in an ascending series of ethanol and xylol followed by embedding in paraffin. Paraffin-embedded tissues were cut into sections of 2 µm thickness and stained with periodic acid Schiff (PAS) using routine protocol. Renal morphology was investigated by light microscopy as described below with the investigator being blinded to the genotype of the mice. Glomerulosclerosis index (GSI) was assessed on PAS-stained paraffin sections at a magnification of 200x. GSI of each animal was derived as the mean of at least 45 randomly sampled glomeruli using a semiquantitative scoring system expressing the severity of glomerulosclerosis on an arbitrary scale from 0 to 4. The glomerular score for individual glomeruli was: grade 0, normal glomerulus; grade 1, presence of mesangial expansion/thickening of the basement membrane; grade 2, mild/moderate segmental hyalinosis/sclerosis involving less than 50% of the glomerular tuft; grade 3, diffuse glomerular hyalinosis/sclerosis involving more than 50% of the tuft; grade 4, diffuse glomerulosclerosis with total tuft obliteration and collapse.

### Immunohistochemistry

Spleens were harvested and frozen in Tissue-Tek OCT Cryomold (Sakura). At a cryotome, 8 µm slices were produced and fixed on Superfrost Plus adhesion Microscopy slides with -20°C acetone for 10 minutes. After rehydration with 1x PBS for 5 minutes, the spleen cryosections were saturated with 10% FCS and 1% Fc-block (2.4G2, hybridoma generated in house) for 30 minutes. The spleen cryosections were stained with Peanut Agglutinin (PNA)-FITC (Vector Laboratories) and anti-IgD-bio (clone 11-26c, eBioscience) for 45 minutes. The slices were washed with 0,05% Tween20 in 1x PBS and subsequently stained with streptavidin-Cy3 (Jackson ImmunoResearch) for 15 minutes. The spleen cryosections were washed with 1x PBS, mounted with Aqua-Poly/Mount (Polysciences) and covered with cover glasses. All spleen cryosections were analyzed the next day with a Axio Scope.A1 fluorescence microscope (Zeiss) while maintaining the same exposure time. Merge of images and analysis were performed with ImageJ.

### Analysis of activation and antibody secretion of *in vitro* stimulated B cells

The spleens of mice at the age of 15-18 weeks were harvested. Generation of single cell suspensions and erythrocyte lysis were performed as described above. 2.5x10^5^ spleen cells were seeded in 1 ml medium (1% heat-inactivated FCS, 1000 U/ml Penicillin-Streptomycin, 1% Non-Essential Amino Acids, 1 mM sodium pyruvate, 200 nM L-glutamine in RPMI-1640 medium) onto 24-well cell culture plates with or without the addition of either 10 µg/ml LPS (Sigma-Aldrich), 10 µg/ml LPS + 10 ng/ml IL-4 (BioLegend), 10 µg/ml LPS + 0,2 µg/ml anti-CD40 antibody (BioLegend) or 10 µg/ml anti-IgM F(ab’)_2_ (Jackson ImmunoResearch). Activation markers were analyzed by flow cytometry after 24 hours by harvesting the cells and staining with anti-CD19-BV421 (clone 1D3, BD Horizon), anti-CD86-FITC (clone GL1, eBioscience) and anti-MHCII-APC (clone AF6-120.1, BioLegend). Antibody concentration in the supernatant was analyzed by ELISA after 6 days of stimulation. Maxisorp plates (96-Well plate; NuncImmuno) were coated with either goat anti-mouse IgM (SouthernBiotech) or goat anti-mouse IgG1 (SouthernBiotech) and saturated with 1% BSA in 1x PBS. The harvested supernatants and IgM or IgG1 standards (both Southern Biotech) were pre-diluted with 0,1% BSA in 1x PBS and added to 96-well plates to produce a 1:3 dilution series. The diluted samples were transferred to the coated ELISA plates. After incubation for 2 hours at 37°C, the ELISA plates were washed and goat anti-mouse IgM-AP (SouthernBiotech) or goat anti-mouse IgG1-AP (SouthernBiotech) were added and incubated for 2 hours at 37°C. After washing, the substrate (4-Nitrophenyl phosphate disodium salt hexahydrate in diethanolamine buffer) was added. The optical density was measured at 405 nm wavelength using a VersaMax microplate reader and analyzed by the Softmax Pro software. Antibody concentration was determined by using the standard curve.

### Statistical analysis

Statistical analyses were performed using GraphPad PRISM software. Outliers were excluded and then the results were tested for normal distribution. For statistical analysis, ANOVA test with Šidák post-hoc test was used in case of normal distribution, whereas the Kruskal-Wallis test with Dunn’s post-hoc test was performed for non-normally distributed samples. Only significant differences in samples are indicated in the figures, non-significant differences are not shown.

## Results

### Germinal center B cells, plasma cells and follicular helper T cells are increased in *Dnase1* ^-/-^ x *Siglecg* ^-/-^ mice, when compared to *Dnase1* ^-/-^ or *Siglecg* ^-/-^ mice.

In order to study the epistatic effects of genes involved in inhibitory signaling and immune complex clearance on autoimmunity, *Siglecg* deficient mice (31) were crossed either with *Dnase1l3* deficient mice (22) or with a newly generated *Dnase1* deficient mouse line. The *Dnase1* deficient mouse line was generated by deleting parts of the *Dnase1* gene by CRISPR/Cas. This led to a 65 bp deletion from intron 2 into exon 3, thereby deleting the splice acceptor site from exon 3 and to predicted frameshift mutations and premature stop codons by potential alternative splicing (**Supplementary Figure 1A**). The 65 bp deletion in genomic DNA was detected by flanking primers and confirmed by sequencing (**Supplementary Figure 1B**). DNase1 activity was tested by incubation of plasmid DNA with urine of WT mice, which led to DNA digestion due to abundant levels of DNase1 in the urine (38). The same assay did not lead to plasmid DNA digestion when performed with the urine of *Dnase1* ^-/-^ mice (**Supplementary Figure 1C**). Since the *Dnase1* ^-/-^ mice were newly generated, we checked their composition of lymphocytes and activation capacity. We noted no changes in splenocyte numbers or in lymphocyte populations. B cells were analyzed in detail. *Dnase1* ^-/-^ mice had normal numbers of total B cells, follicular B cells, marginal zone B cells in the spleen and B1a cells in the peritoneum (**Supplementary Figure 2A**). When stimulated with various stimuli, B cells of *Dnase1* ^-/-^ mice had a normal upregulation of activation markers (**Supplementary Figure 2B**) and normal antibody production (**Supplementary Figure 2C**). *Dnase1* ^-/-^ mice and all other mouse lines used in this study were on a C57BL/6 background. All mice were aged and analyzed at 24 weeks, 36 weeks, 48 weeks and 60 weeks of age. At 24 and 36 weeks of age no clear differences in autoimmune parameters were observed between the various genotypes, therefore the results from 48 week and 60 week-old animals are presented here.

**Figure 1:**
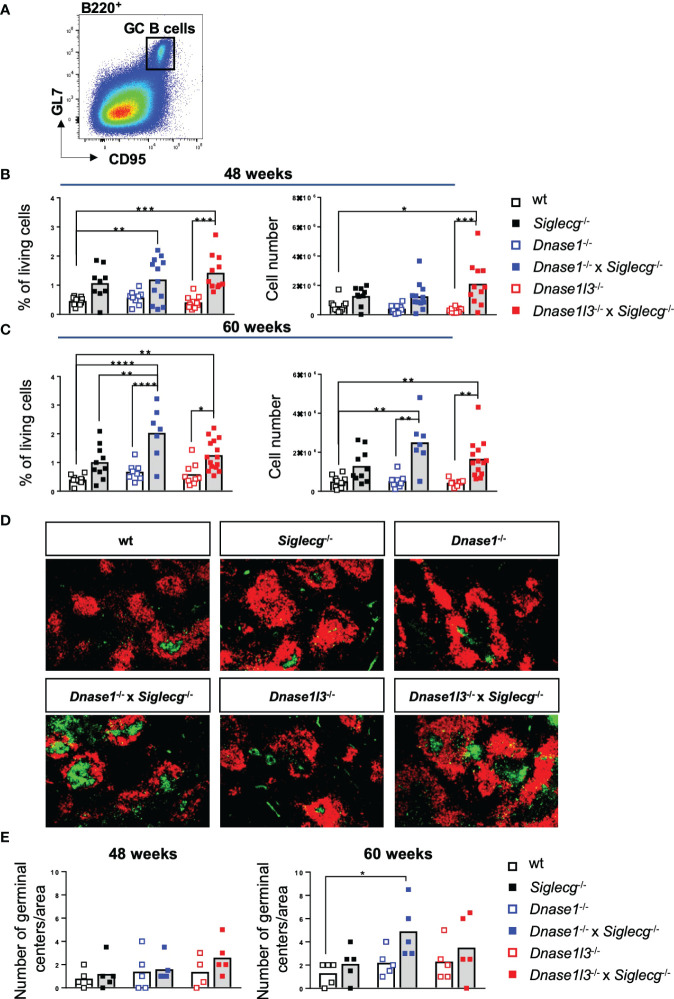
Germinal center B cells are increased in *Dnase1* ^-/-^ x *Siglecg* ^-/-^ mice, compared to *Dnase1^-/-^* or *Siglecg^-/-^* mice. **(A)** Gating strategy for murine GC B cells in aging mice. Splenic cells were pre-gated on single cells, living cells and lymphocytes. Subsequently, B220^+^ CD95^+^ GL7^+^ cells were defined as GC B cells. **(B)** Quantification of GC B cells of 43-48 week-old mice. The left graph shows the percentage of GC B cells among living cells and the right graph shows the total cell number of GC B cells. Every point represents one mouse, the bars represent the mean values and data are generated from 9-12 mice per genotype within 9 independent experiments. **(C)** Quantification of GC B cells of 60 week- old mice. The left graph shows the percentage of GC B cells among living cells and the right graph shows the total cell number of GC B cells. Every point represents one mouse, the bars represent the mean values and data are generated from 7-15 mice per genotype within 7 independent experiments. **(D)** Germinal centers were stained with PNA-FITC (green) and B cell follicles with anti-IgD-Cy3 (red) on spleen cryosections and analyzed with a fluorescence microscope (50x magnification). Shown are representative images of 60 week old mice of indicated genotypes. **(E)** Quantification of the number of germinal centers per area in spleen cryosections of 48 week and 60 week old mice. Every point represents the average number of germinal centers of two analyzed areas per spleen cryosection of one mouse, the bars represent the mean values and data are generated from 4-5 mice per genotype. For statistical analysis, ANOVA test with Šidák post-hoc test was used in case of normal distribution, whereas the Kruskal-Wallis test with Dunn’s post-hoc test was performed for non-normally distributed samples. p<0.05(*), p<0.01 (**), p<0.001 (***), p<0.0001 (****).

Spontaneous development of germinal centers is a hallmark in SLE formation in humans and mice (39). Therefore, GC B cell percentages and numbers were determined for *Dnase1* ^-/-^ x *Siglecg* ^-/-^, *Dnase1l3* ^-/-^ x *Siglecg* ^-/-^ mice, the corresponding *Siglecg* ^-/-^, *Dnase1* ^-/-^ and *Dnase1l3* ^-/-^ mice as well as wt mice. *Siglecg* deficiency alone tended to have an enhancing influence on GC B cell numbers in aging mice since both percentage and total GC B cell numbers were increased in *Siglecg* ^-/-^ mice compared to the wt mice (**Figures 1A–C**). In contrast, single-deficient *Dnase1* ^-/-^ or *Dnase1l3* ^-/-^ mice did not show this tendency (**Figures 1A–C**). However, both *Dnase1* ^-/-^ x *Siglecg* ^-/-^ and *Dnase1l3* ^-/-^ x *Siglecg* ^-/-^ double-deficient mice show significantly increased GC B cell numbers at most time points, when compared to single-deficient *Dnase1* ^-/-^ or *Dnase1l3* ^-/-^mice. However, only *Dnase1* ^-/-^ x *Siglecg* ^-/-^ mice showed a relative increase of GC B cells at 60 weeks, when compared to *Siglecg* ^-/-^ mice (**Figure 1C**). Similar results were also found in lymph nodes (**Supplementary Figures 3A–C**). Analysis of the GC structure by histology revealed a significantly higher number of GCs only in *Dnase1* ^-/-^ x *Siglecg* ^-/-^ mice compared to wt mice, but it is more difficult to quantify small differences in GC numbers by histology (**Figures 1D, E**). We did not detect differences in the sizes of individual GCs in the different mutant mice. Thus, the *Siglecg* deficiency seems to be mainly responsible for the increase of GC B cell numbers and the *Dnase1* deficiency enhances this phenotype.

**Figure 2:**
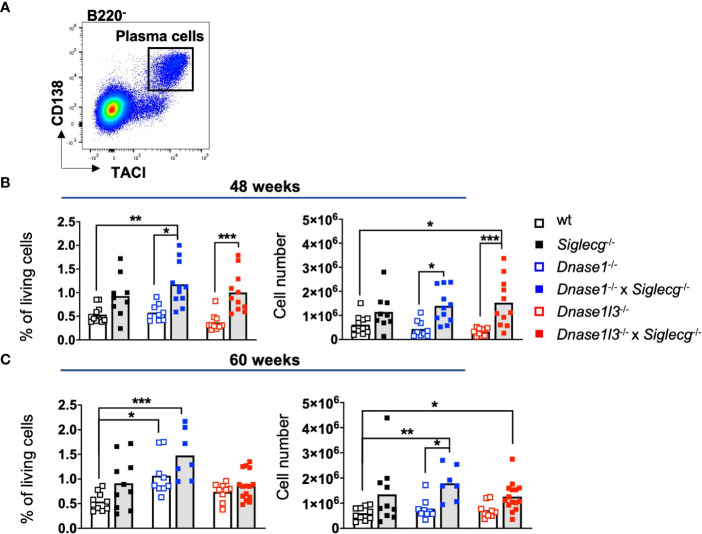
Plasma cells are increased in *Siglecg* ^-/-^, *Dnase1* ^-/-^ x *Siglecg* ^-/-^ and *Dnase1l3* ^-/-^ x *Siglecg* ^-/-^ mice, without a further increase in double-deficient mice. **(A)** Gating strategy for splenic plasma cells in aging mice. Cells were pre-gated on single cells, living cells and lymphocytes. Subsequently, B220^-^ TACI^+^ CD138^+^ cells were defined as plasma cells. **(B)** Quantification of plasma cells of 43-48 week old mice. On the left, the percentage of plasma cells among living cells is shown. The total plasma cell numbers are depicted on the right. Every point represents one mouse, the bars represent the mean values and data are generated from 9-11 mice per genotype within 9 independent experiments. **(C)** Quantification of plasma cells of 60 week old mice. On the left, the percentage of plasma cells among living cells is shown. The total cell numbers are depicted on the right. Every point represents one mouse, the bars represent the mean values and data are generated from 7-15 mice per genotype within 7 independent experiments. For statistical analysis, ANOVA test with Šidák post-hoc test was used in case of normal distribution, whereas the Kruskal-Wallis test with Dunn’s post-hoc test was performed for non-normally distributed samples. p<0.05(*), p<0.01 (**), p<0.001 (***).

Beside increased numbers of GC B cells due to spontaneous germinal center formation, plasma cells producing pathogenic autoantibodies contribute to SLE formation and are therefore an established marker (40). Therefore, a flow cytometry analysis of plasma cells in the spleen (**Figures 2A–C**) or in the bone marrow (**Supplementary Figures 3D–F**) was performed. Similar to GC B cells, plasma cell percentages and numbers tended to be increased in *Siglecg* ^-/-^ mice at the age of both 48 weeks and 60 weeks in the spleen (**Figures 2B, C**). In contrast, single-deficient *Dnase1* ^-/-^ or *Dnase1l3* ^-/-^ mice did not show this general tendency (**Figures 2B, C**). Again, both *Dnase1* ^-/-^ x *Siglecg* ^-/-^ and *Dnase1l3* ^-/-^ x *Siglecg* ^-/-^ double-deficient mice showed significantly increased plasma cell numbers at most time points in the spleen, when compared to single-deficient *Dnase1* ^-/-^ or *Dnase1l3* ^-/-^ mice, but not in comparison to *Siglecg* ^-/-^ mice (**Figures 2B, C**). Similar tendencies of increased plasma cell numbers in double-deficient mice were also found in the bone marrow, albeit with no significant differences (**Supplementary Figures 3E, F**). Again, the *Siglecg* deficiency seems to be mainly responsible for the increase of plasma cell numbers in aging mice.

**Figure 3:**
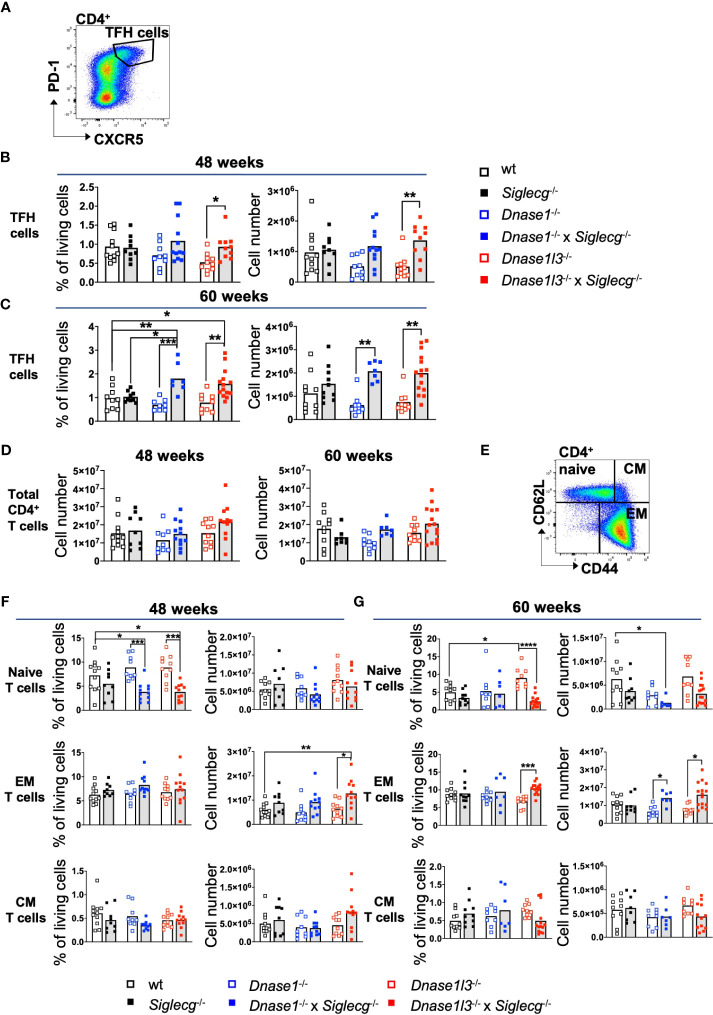
T-follicular helper cells are increased in *Dnase1^-/-^ x Siglecg^-/-^* mice, compared to *Dnase1^-/-^* or *Siglecg^-/-^* mice. **(A)** Gating strategy for murine TFH cells in aging mice. Splenocytes were pre-gated on single cells, living cells and lymphocytes. Subsequently, CD4^+^ CXCR5^+^ PD-1^+^ cells were defined as TFH cells. **(B)** Quantification of TFH cells of 43-48 week old mice. On the left, the percentage of TFH cells from the living cells is shown. The total cell numbers of TFH cells are depicted on the right. Every point represents one mouse, the bars represent the mean values and data are generated from 9-12 mice per genotype within 9 independent experiments. **(C)** Quantification of TFH cells of 60 week old mice. On the left, the percentage of TFH cells from the living cells is shown. The total cell numbers of TFH cells are depicted on the right. Every point represents on mouse, the bars represent the mean values and data are generated from 7-15 mice per genotype within 7 independent experiments. **(D)** Quantification of CD4^+^ T cells of 43-48 week old mice or 60 week old mice. The graphs show the total cell number. Every point represents one mouse, the bars represent the mean values and data are generated from 9-12 mice per genotype within 9 independent experiments (48 weeks) or 7-15 mice per genotype within 7 independent experiments (60 weeks). **(E)** Gating strategy for murine CD4^+^ naïve and memory T cell populations from the spleen in aging mice. Cells were pre-gated on single cells, living cells and lymphocytes. Subsequently, CD4^+^ T cells were distinguished into naïve (CD62L^+^, CD44^low^), effector memory (EM; CD62L^low^, CD44^+^) and central memory (CM; CD62L^+^, CD44^+^) T cells based on their CD62L and CD44 expression. **(F)** Quantification of CD4^+^ naïve, EM T cells and CM T cells of 43-48 week old mice. In each case, the left graph shows the percentage of naïve, EM T cells or CM T cells from living cells and the right graph shows the total cell numbers. Every point represents one mouse, the bars represent the mean values and data are generated from 9-12 mice per genotype within 9 independent experiments. **(G)** Quantification of CD4^+^ naïve, EM T cells and CM T cells of 60 week old mice. In each case, the left graph shows the percentage of naïve, EM T cells or CM T cells from living cells and the right graph shows the total cell numbers. Every point represents one mouse, the bars represent the mean values and data are generated from 7-15 mice per genotype within 7 independent experiments. For statistical analysis, ANOVA test with Šidák post-hoc test was used in case of normal distribution and for non-normally distributed samples, the Kruskal-Wallis test with Dunn’s post-hoc test was performed. p<0.05(*), p<0.01 (**), p<0.001 (***), p<0.0001 (****).

Alongside germinal center B cells, numbers of follicular helper T cells (TFH) were analyzed in this study to determine spontaneous germinal center formation. For this purpose, TFH cell numbers in the spleen of *Dnase1* ^-/-^ x *Siglecg* ^-/-^ and *Dnase1l3* ^-/-^ x *Siglecg* ^-/-^ as well as the corresponding single knock out mice and wt mice were investigated (**Figures 3A–C**). While at all time points no clear difference in TFH cell numbers of single-KO mice to wt controls could be observed, *Dnase1* ^-/-^ x *Siglecg* ^-/-^ or *Dnase1l3* ^-/-^ x *Siglecg* ^-/-^ mice showed significantly increased TFH cell numbers at most time points, when compared to single-deficient *Dnase1* ^-/-^ or *Dnase1l3* ^-/-^ mice. *Dnase1* ^-/-^ x *Siglecg* ^-/-^ mice also showed a relative increase of TFH cell numbers, when compared with *Siglecg* ^-/-^ mice at 60 weeks of age (**Figures 3B, C**). In contrast to these changes of TFH cells, numbers of total CD4 T cells were not changed in *Dnase* x *Siglecg* ^-/-^ mice (**Figure 3D**).

A further phenotype of SLE is a higher transition of naïve T cells to effector memory (EM) T cells and central memory (CM) T cells. To examine whether the ratio of naïve and activated memory T cells is altered in *Dnase1* ^-/-^, *Dnase1* ^-/-^ x *Siglecg* ^-/-^, *Dnase1l3* ^-/-^ and *Dnase1l3* ^-/-^ x *Siglecg* ^-/-^ mice, *Siglecg* ^-/-^ and wt mice, splenic T cells were analyzed *via* flow cytometry (**Figure 3E**). Indeed, the percentages of CD4^+^ naïve T cells in 48 week-old *Dnase1* ^-/-^ x *Siglecg* ^-/-^ and *Dnase1l3* ^-/-^ x *Siglecg* ^-/-^ mice were significantly reduced compared to wt and *Dnase* single-KO mice (**Figure 3F**, left). However, this is neither visible in total cell numbers nor at 60 weeks of age (**Figures 3F, G**). With respect to the CD4^+^ memory T cells, the CM T cell numbers were comparable between the different genotypes, whereas EM T cell numbers were significantly increased in *Dnase1l3* ^-/-^ x *Siglecg* ^-/-^ mice compared to wt and *Dnase1l3 single-KO* mice (**Figures 3F, G**). Similar tendencies are detectable for CD8^+^ T cell subsets (**Supplementary Figure 4**).

### Autoantibodies are strongly increased in *Siglecg* ^-/-^ x *Dnase1l3* ^-/-^ mice

**Figure 4:**
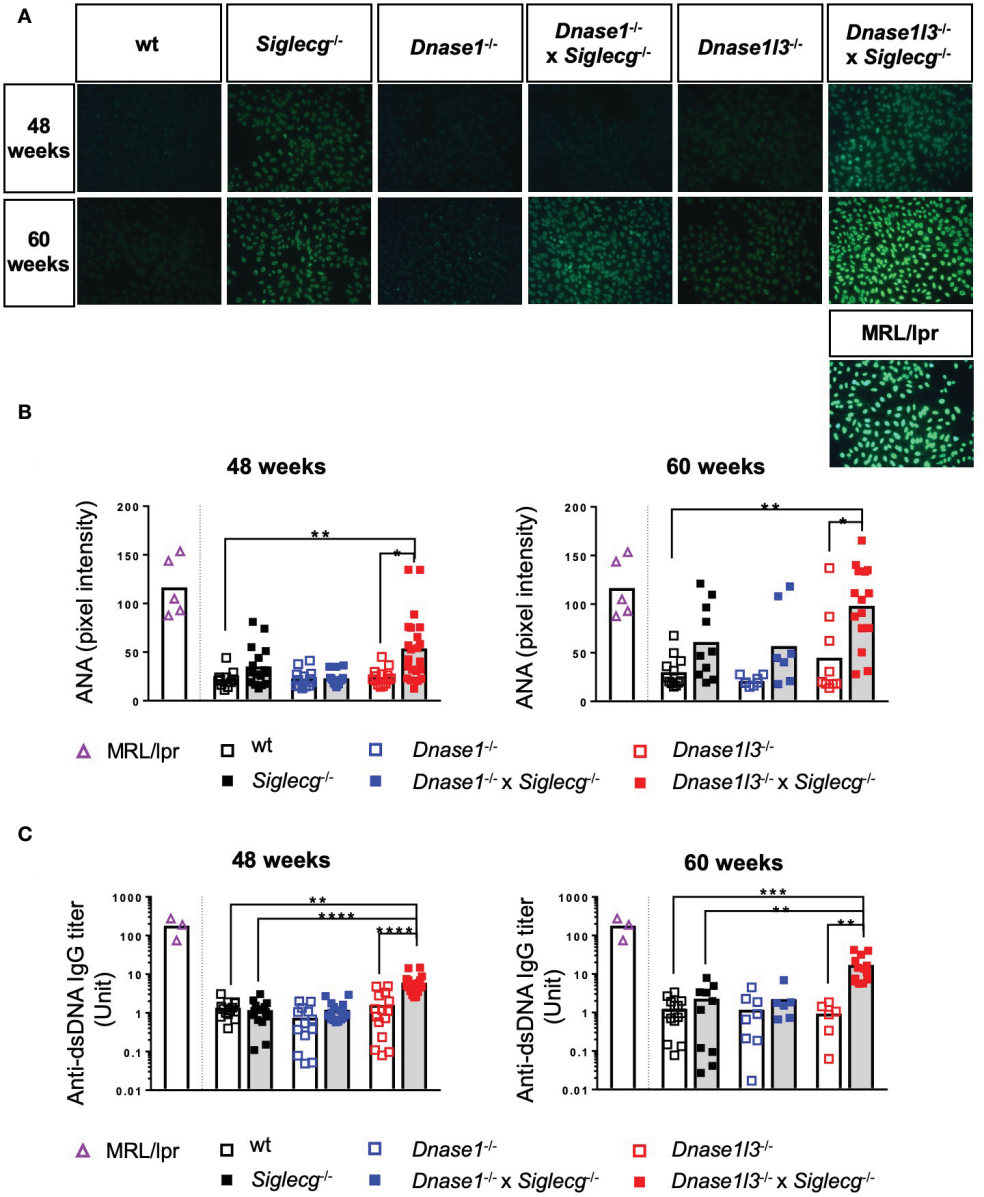
*Dnase1l3* ^-/-^ x *Siglecg* ^-/-^ mice develop increased levels of autoantibodies with age compared to *Dnase1l3* ^-/-^ and *Siglecg* ^-/-^ mice. At 43-48 weeks and 60 weeks of age sera of naïve mice were taken and analyzed for anti-nuclear antibodies **(A, B)** and anti-double stranded DNA (dsDNA) antibodies **(C)**. Sera of sick MRL/lpr mice (around 20 weeks of age) serve as a positive control. **(A)** Detection of anti-nuclear antibodies (ANA) on HEP2-slides. Fluorescence images show representative pictures from mice of the tested genotypes at 48 and 60 weeks of age. The images were taken with the identical exposure time and in 200x magnification. **(B)** Quantification of fluorescence intensity of anti-mouse IgG-AF488 antibodies bound to ANAs in sera of 48 week and 60 week old mice. Every point represents one mouse, the bars represent the mean values and data are generated from 12-22 mice (48 weeks) or 7-15 mice (60 weeks) per genotype within 4 independent experiments. **(C)** Quantification of anti-dsDNA antibodies in sera of aging mice. Antibody titers of 48 week and 60 week old mice were determined by ELISA. The serum of NZB/W mice served as standard and therefore antibody titers are expressed as arbitrary units. Every point represents one mouse, the bars represent the mean values and data were generated from 13-20 mice (48 weeks) or 6-14 mice (60 weeks) per genotype within 3 independent experiments. For statistical analysis, the Kruskal-Wallis test with Dunn’s post-hoc test was performed. p<0.05(*), p<0.01 (**), p<0.001 (***), p<0.0001 (****).

Next, autoantibodies directed against nuclear components (ANAs) and double stranded DNA (anti-dsDNA) were tested in aging mice. First, sera of naïve aging mice were tested for the presence of ANAs. Both exemplary images and quantification showed that a deficiency in *Siglecg* leads to more ANAs compared to wt mice at 48 weeks and 60 weeks, although this increase was not significant (**Figures 4A, B**). There was no increase of ANAs detected in *Dnase1* ^-/-^ mice and a trend towards higher ANA levels in *Dnase1l3* ^-/-^ mice at 60 weeks. *Dnase1l3* ^-/-^ x *Siglecg* ^-/-^ mice showed a significant increase of ANAs, when compared to *Dnase1l3* ^-/-^ or wt mice (**Figures 4A, B**). As a positive control, sera of sick, 4-5 months old MRL/*lpr* mice are shown. At 60 weeks, *Dnase1l3* ^-/-^ x *Siglecg* ^-/-^ mice reached ANA levels which were comparable to sick MRL/*lpr* mice.

Additionally, anti-dsDNA antibodies in the serum of the aging mice were quantified *via* ELISA. No increases of anti-dsDNA antibodies were detected in any single-KO mice, when compared to wt controls (**Figure 4C**). A substantial increase in anti-dsDNA IgG autoantibodies was detected in the serum of naive *Dnase1l3* ^-/-^ x *Siglecg* ^-/-^ compared to wt mice and to *Siglecg* ^-/-^ and *Dnase1l3* ^-/-^ single knockout mice, however (**Figure 4C**). This increase was already present at the age of 48 weeks and demonstrates epistatic effects of both deleted genes on the development of anti-dsDNA antibodies. Interestingly, in double-deficient *Dnase1* ^-/-^ x *Siglecg* ^-/-^ mice no such epistatic effect is observable (**Figure 4C**).

### Histological analysis of the kidneys revealed glomerulonephritis in *Dnase1^-/-^* x *Siglecg^-/-^* and *Dnase1l3^-/-^* x *Siglecg^-/-^* mice

**Figure 5:**
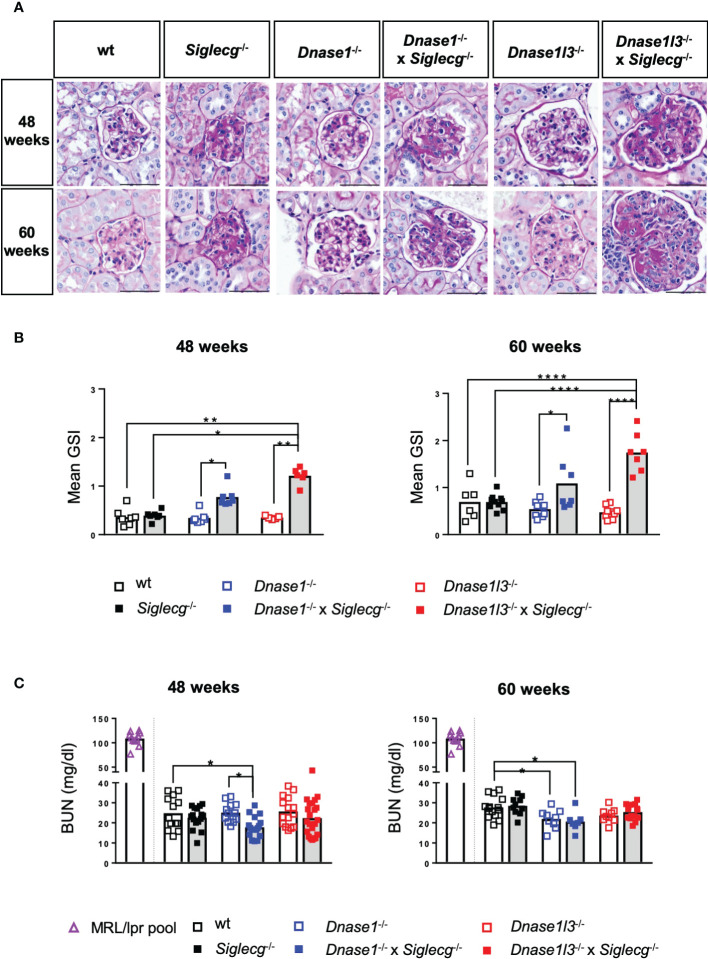
Histological analysis of the kidneys revealed glomerulonephritis in *Dnase1^-/-^ x Siglecg^-/-^* and more severe in *Dnase1l3^-/-^ x Siglecg^-/-^* mice. **(A)** Histological evaluation of the glomerulosclerosis index (GSI). Representative glomeruli from periodic acid-Schiff (PAS)-stained kidney sections of 48 week and 60 week old mice. Scale bar represents 50 µm. **(B)** Assessment of glomerulosclerosis index (GSI). Kidneys from mice of the respective genotypes were scored semi-quantitatively for glomerular changes observed in lupus nephritis. Every point represents the mean GSI from one mouse and data were generated from 6-7 (48 weeks) and 6-10 (60 weeks) per genotype. **(C)** Blood urea nitrogen (BUN) levels in the serum were quantified. A serum pool of MRL/lpr mice (one pool, around 20 weeks of age) was included as a positive control (purple). Every point represents one mouse, the bars represent the mean values and data are generated from 13-22 mice (48 weeks) or 7-15 mice (60 weeks) per genotype within 3 independent experiments. For statistical analysis, ANOVA test with Šidák post-hoc test was used in case of normal distribution and for non-normally distributed samples, the Kruskal-Wallis test with Dunn’s post-hoc test was performed. p<0.05(*), p<0.01 (**), p<0.0001 (****).

Because deposition of immune complexes in the kidney frequently occurs during the course of lupus disease and can lead to severe renal damage, we examined the kidneys histopathologically and evaluated them for glomerular changes including mesangial matrix expansion, glomerular size and cell counts using periodic acid Schiff (PAS) stain. Examples of PAS-stained glomeruli of the various mice genotypes are shown in **Figure 5A**. While the glomeruli of *Dnase1* ^-/-^ and *Dnase1l3* ^-/-^ exhibited a normal PAS-positive mesangium, the mesangium of *Siglecg* ^-/-^ mice appeared slightly widened. This change was more pronounced in the two double-knockout mice, with the *Dnase1l3* ^-/-^ x *Siglecg* ^-/-^ animals exhibiting additional mesangial proliferation, particularly at 60 weeks of age, which also resulted in enlarged glomeruli and narrowing of capillary volume (**Figure 5A**), indicating progressed glomerular injury.

When quantified with a mean glomerulosclerosis index (GSI), single-KO mice did not show significant alterations. However, both *Dnase1* ^-/-^ x *Siglecg* ^-/-^ and *Dnase1l3^-/-^* x *Siglecg^-/-^* mice revealed a significantly increased mean glomerulosclerosis index compared to *Dnase1* ^-/-^ and *Dnase1l3* ^-/-^ single knockout mice (**Figure 5B**). The GSI of *Dnase1l3^-/-^* x *Siglecg^-/-^* mice was higher than the GSI of *Dnase1* ^-/-^ x *Siglecg* ^-/-^ mice, at both time points. Histopathological changes in the kidney were confined to the glomerulus in investigated mice, with only *Dnase1l3* ^-/-^ x *Siglecg* ^-/-^ showing mild tubulointerstitial changes with tubule dilatations and an increased inflammatory response. The renal vessels showed no pathological changes in all mice.

In addition, the blood urea nitrogen (BUN) content was examined in aging *Dnase1* ^-/-^, *Dnase1* ^-/-^ x *Siglecg* ^-/-^, *Dnase1l3* ^-/-^ and *Dnase1l3* ^-/-^ x *Siglecg* ^-/-^ mice, *Siglecg* ^-/-^ mice and wt mice to examine the consequences of kidney damage. As depicted in **Figure 5C**, all tested genetically-modified mice showed no signs of elevated blood urea nitrogen levels, compared to wt controls, neither at 48 weeks of age nor at 60 weeks. The BUN levels found were between 20 to 30 mg/dl. This was in contrast to sick MRL/*lpr* animals, analyzed as a positive control, with high BUN values around 100 mg/dl. In conclusion, both *Dnase1* ^-/-^ x *Siglecg* ^-/-^, as well as *Dnase1l3* ^-/-^ x *Siglecg* ^-/-^ aging mice developed glomerulonephritis, with a more severe disease score detected in the latter mouse line.

## Discussion

Predisposition to SLE is suggested to originate from epistatic effects of several susceptibility genes. We examined here the contribution of genes which are involved in separate biological processes that have been identified to correlate with SLE susceptibility. We examined two DNase genes, involved in immune complex clearance, in combination with a gene coding for an inhibitory receptor in B cells. Our results show that a combination of deficiencies in these two processes can lead to enhanced lupus-like symptoms in mouse models. The two members of the DNase1 family were contributing to varying extent to this susceptibility, however. While the combination of mutations in *Dnase1* and *Siglecg* led to increased germinal centers, without affecting autoantibody levels, mutations in *Dnase1l3* and *Siglecg* led to strongly increased levels of autoantibodies. Both combinations of gene deficiencies led to glomerular damage in the kidney, however with a higher glomerular damage score in *Dnase1l3* ^-/-^ x *Siglecg* ^-/-^ mice.

Mutations in the *DNASE1* are associated with SLE in humans (18) and SLE development was also reported in mouse models (15, 16, 21). In contrast to these previous reports, our newly generated *Dnase1* ^-/-^ mouse line does not show any indications of autoantibodies, enlarged populations of GC or plasma cells, nor kidney alterations, up to the age of 60 weeks. The deletion in this line is introduced into intron 2 and exon 3 and therefore does not affect the neighboring *Trap1* gene. In the other mouse line with a *Dnase1* mutation without affecting *Trap1*, a rather mild increase of autoantibodies and nephritis score is reported from 9 months onwards (21). Mutations in the *DNASE1L3* are associated with SLE in three independent families (22, 23). In the mouse, autoantibodies and kidney alterations were reported in two independent *Dnase1l3* ^-/-^ mouse lines (15, 22). However, a strong increase of anti-dsDNA antibodies was only seen, when an additional gene mutation, the *FcgrIIb*-deficiency was present as well (22).

Aging *Siglecg* ^-/-^ mice show increased GC B cells and plasma cells and a mild increase of autoantibodies (31). Also for *Siglecg*, an epistatic genetic effect was observed when the *Siglecg* deficiency was combined with other deficiencies of B cell inhibitory receptors, i.e. with *Cd22* or with *FcgrIIb* (24, 31). The human Siglec-G orthologue *SIGLEC10* was so far not detected as a monogenic cause for SLE, nor identified in GWAS studies. However, genes in the inhibitory pathway of *SIGLEC10*, such as *LYN*, *BLK* or *BANK1* were identified in SLE GWAS screens (8, 41). Thus, for all of the three examined alleles in this study, it seems that single mutations lead to rather weak lupus-like symptoms, whereas higher autoantibody titers and strong glomerulonephritis are present in combinations of these mutations.

However, there were notable differences between the two *Dnase*- deficient mouse lines. While a combination of the *Siglecg*-deficient allele with the *Dnase1*-deficient allele led to increased GC B cells and TFH cells, when compared to the *Siglecg*-deficiency alone, only *Dnase1l3* ^-/-^ x *Siglecg* ^-/-^ mice developed high levels of anti-DNA and anti-nuclear antibodies. It has been shown previously that anti-DNA antibodies can evolve from non-autoreactive progenitors in GCs (42). A higher GC activity would be indicated by an increased number of GC B cells and TFH cells in aging mice, as observed here. Higher number of GC B cells and plasma cells have been observed in *Siglecg^-/-^* mice before (31). Thus, the increased GC cell expansion of *Dnase1* ^-/-^ mice, when combined with *Siglecg*-deficiency, indicates an involvement of the GC in autoantibody formation in this case. However, it has been demonstrated that DNase1l3 is more efficient than DNase1 in removal of apoptotic microparticles than DNase1 (15). DNase1 prefers to cut “naked” DNA, whereas DNase1l3 has a high cleavage activity for nuclear DNA, in its native chromatin form (43). This is relevant for clearance of nuclear material, including DNA, in chromatin immune complexes. Furthermore, DNase1l3 is secreted to a high extent by antigen-presenting cells, such as DCs or Kupffer cells in the liver. It was proposed that this secretion by antigen-presenting cells may be used as a protection of activation of TLRs by chromatin-containing immune complexes (22). Both mechanisms can explain, why Dnase1l3 is more important in preventing autoantibody production than DNase1, which is mainly found in the gastro-intestinal tract and expressed in high levels in the kidney (17).

Lupus nephritis is a severe clinical manifestation in SLE. In this respect it was interesting that glomerular changes in the kidney were moderate in *Dnase1^-^* ^/-^ x *Siglecg* ^-/-^ mice, while they were quite severe in *Dnase1l3* ^-/-^ x *Siglecg* ^-/-^ mice. Glomerulonephritis is caused by immune complex deposition in the glomeruli that can cause glomerular changes and damage, leading ultimately to nephritis. The difference in severity in glomerular changes may most likely be explained by the specificity of DNase1l3 for DNA in chromatin, which is part of immune complexes containing nuclear material in the kidney, as discussed above. Despite a significant exacerbation of glomerular damage in *Dnase1l3* ^-/-^ x *Siglecg* ^-/-^ mice, elevated levels of BUN in the blood are not detectable. However, BUN is known to be a less sensitive marker of early nephritis as it depends on multiple factors (44). Although pronounced histopathological changes were detected especially in the *Dnase1l3* ^-/-^ x *Siglecg* ^-/-^ animals, they were less pronounced compared with MRL/lpr and therefore did not result in a detectable increase in BUN.

In conclusion, we have shown epistatic interactions of genes which are involved in inhibitory signaling or in immune complex clearance in the development of SLE. While deficiencies of the individual genes caused only mild symptoms of autoimmunity, a combination of two gene deficiencies, resulting in consequences in both of these different biological processes, had a strong influence on SLE development in mouse models. This is reflecting the situation in human SLE, which is mostly a polygenetic disorder. The loss of the inhibitory receptor in combination with the loss of DNase1l3 had a more severe disease progression than the combination with the loss of DNase1. This is likely due to the difference in enzymatic activities, as only DNase1l3 has a significant DNA degradation capacity on nuclear particles containing genomic DNA in its native chromatin form, as resulting from apoptotic processes. Our study thus emphasizes the importance of synergistic effects of genes with distinct functions that can amplify the severity of SLE.

## Data availability statement

The original contributions presented in the study are included in the article/**Supplementary Material**. Further inquiries can be directed to the corresponding author.

## Ethics statement

The animal study was reviewed and approved by Regierung von Unterfranken, Würzburg.

## Author contributions

MK did experiments and wrote manuscript. MS did experiments and wrote manuscript. CB did experiments. DR did experiments. CD did experiments and discussed results. TW did experiments and discussed results. LN supervised the study and wrote manuscript. All authors contributed to the article and approved the submitted version.
