# Supplementary material for: Epistatic effects of Siglec-G and DNase1 or DNase1l3 deficiencies in the development of systemic lupus erythematosus
Source: Front Immunol. 2023 Mar 8;14:1095830. doi: 10.3389/fimmu.2023.1095830 (PMC10030676; doi:10.3389/fimmu.2023.1095830)
Supplement: Supplementary file 1 [file DataSheet_1.pdf]

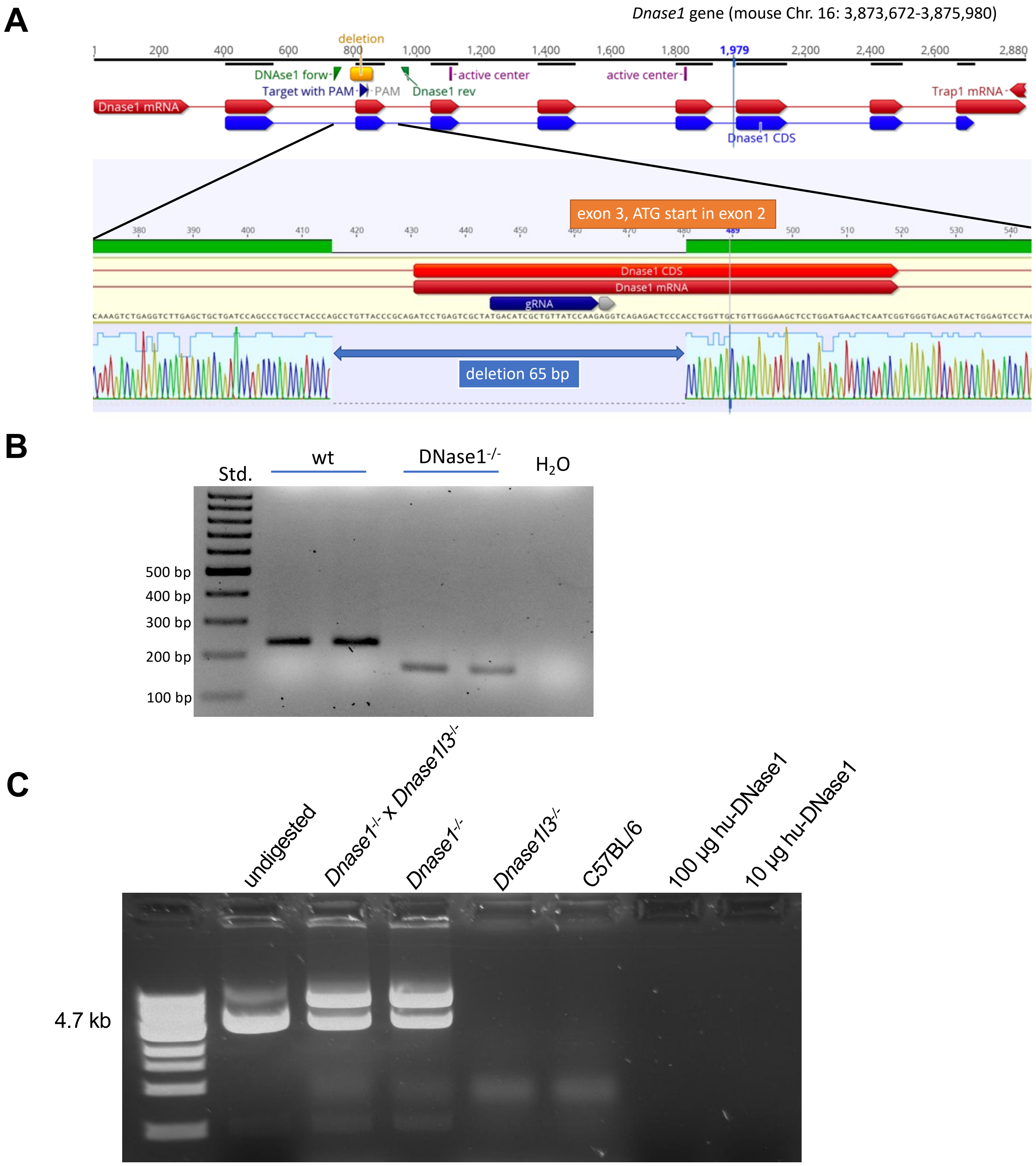

Supplementary Figure 1:

Generation of *Dnase1*$^{-/-}$ mice by CRISPR/Cas9 mediated mutagenesis in JM8A3 embryonic stem (ES) cells from C57BL/6N origin.

A) Displayed is the *Dnase1* gene with its exon/ intron structure. The binding site of the *Dnase1*-specific gRNA 5´ TGACATCGCTGTTATCCAAG 3´ in exon 3 of the wt allele is shown in violet. CRISPR-Cas9 induced double-strand break repair created a 65 bp deletion (yellow) overlapping from intron 2 into exon 3, thereby deleting the splice acceptor site from exon 3 (shown at the bottom). The deletion does not allow splicing into exon 3, and potential alternative splicing into exons 4, 5 or 6 containing the active sites for Dnase1 enzymatic activity would lead to frameshift mutations and premature stop codons.

B) PCR of genomic DNA of WT and *Dnase1*$^{-/-}$ mice with DNAase1forw and DNAase1rev primers, showing the expected bands of 230 bp and 165 bp, respectively.

C) Functional verification of the *Dnase1* knockout in *Dnase1*$^{-/-}$ mice via digestion of a 4.7 kb plasmid by DNases in the urine of mice. The plasmid was incubated with urine of *Dnase1*$^{-/-}$ x *Dnase1l3*$^{-/-}$ mice, *Dnase1*$^{-/-}$ mice or C57BL/6 mice. As further controls served undigested DNA and DNA digested by either 10 µg or 100 µg of recombinant human DNase1. The DNA integrity was subsequently tested via gel electrophoresis.

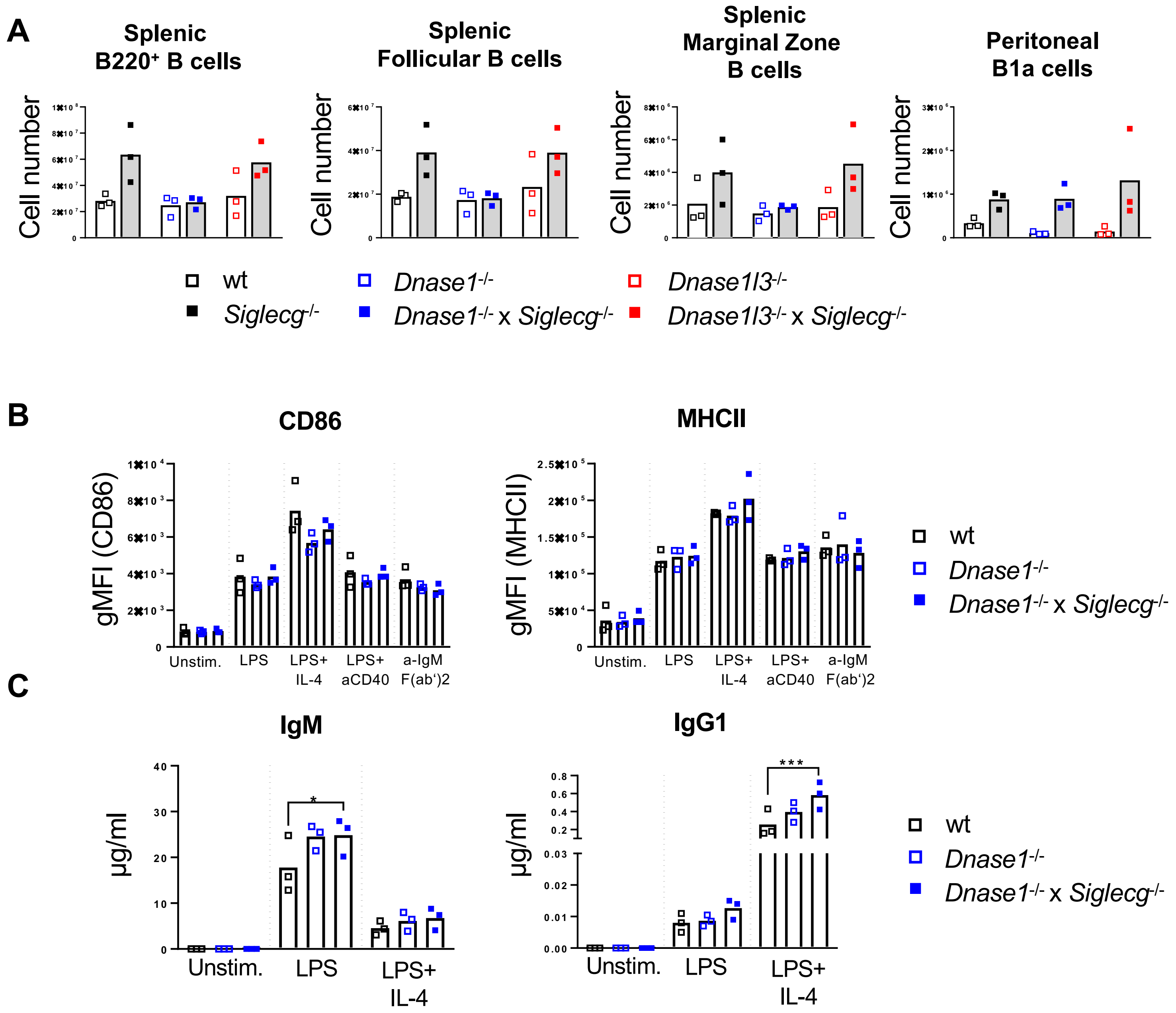

Supplementary Figure 2:

Dnase1$^{-/-}$ mice have normal B cell populations, show a normal upregulation of activation markers and normal antibody secretion after stimulation in vitro.

(A) Quantification of splenic B220^{+} B cells, follicular B cells (B220^{+} CD21^{low} CD23^{+}), marginal zone B cells (B220^{+} CD21^{high} CD23^{low}) and peritoneal B1a (B220^{low} CD5^{+}) B1a cells of 15-18 week old mice. The graphs show the total cell number of indicated cell types. Every point represents one mouse, the bars represent the mean values and data are generated from 3 mice per genotype within 3 independent experiments.

(B) Quantification of CD86 expression (left graph) and MHCII expression (right graph) on B cells after *in vitro* stimulation of splenic cells for 24 hours with either no stimulant, 10 µg/ml LPS, 10 µg/ml LPS + 10 ng/ml IL-4, 10 µg/ml LPS + 0,2 µg/ml anti-CD40 antibody or 10 µg/ml anti-IgM F(ab')$_2$. The graphs show the geometric mean fluorescence intensity of CD86 or MHCII. Every point represents one mouse, the bars represent the mean values and data are generated from 3 mice per genotype with an age of 15-18 weeks.

(C) Quantification by ELISA of IgM (left graph) or IgG1 (right graph) antibody secretion in the supernatant of *in vitro* stimulated spleen cells. Stimulation was performed for 6 days with either no stimulant, 10 µg/ml LPS or 10 µg/ml LPS + 10 ng/ml IL-4. The graphs show the concentration of IgM or IgG1 in the supernatant. Every point represents one mouse, the bars represent the mean values and data are generated from 3 mice per genotype with an age of 15-18 weeks.

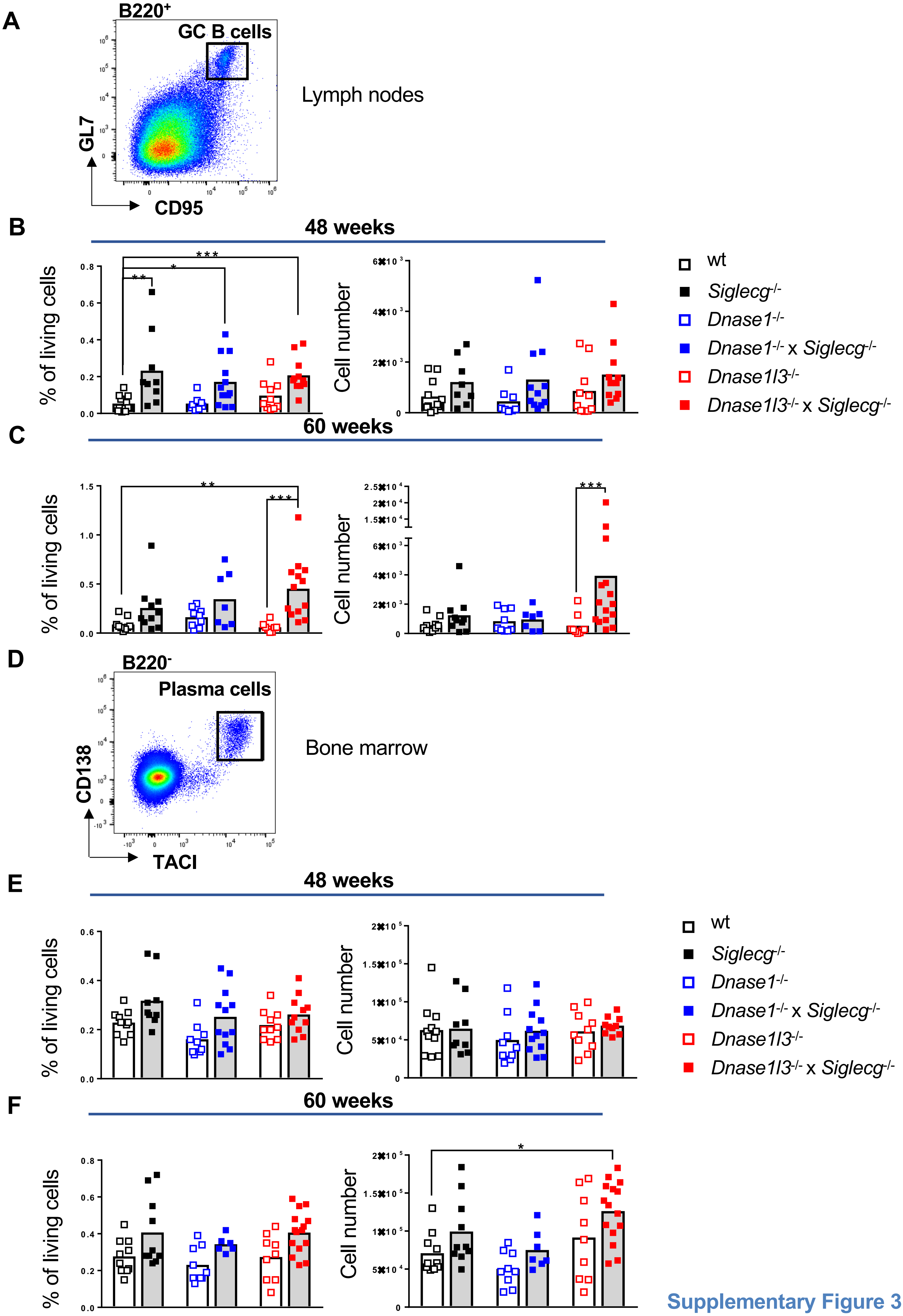

Supplementary Figure 3

Supplementary Figure 3:

Murine lymph node cells were stained with fluorescently labeled antibodies for GC B cells and bone marrow cells were stained for plasma cells to analyze them via flow cytometry. Cells of the following genotypes were investigated: WT (black), *Siglecg*$^{-/-}$ (black painted), *Dnase1*$^{-/-}$ (blue), *Dnase1*$^{-/-}$ x *Siglecg*$^{-/-}$ (blue painted), *Dnase1l3*$^{-/-}$ (red), *Dnase1l3*$^{-/-}$ x *Siglecg*$^{-/-}$ (red painted).

(A) Gating strategy for murine GC B cells in the lymph nodes. Cells were pre-gated on single cells, living cells and lymphocytes. Subsequently, B220^{+} CD95^{+} GL7^{+} cells were defined as GC B cells.

(B) Quantification of GC B cells in the lymph nodes of 43-48 week old mice. The left graph shows the percentage of GC B cells among living cells and the right graph shows the total cell number of GC B cells. Every point represents one mouse, the bars represent the mean values and data are generated from 9-12 mice per genotype within 9 independent experiments.

(C) Quantification of GC B cells in the lymph nodes of 60 week old mice. The left graph shows the percentage of GC B cells among living cells and the right graph shows the total cell number of GC B cells. Every point represents one mouse, the bars represent the mean values and data are generated from 7-15 mice per genotype within 7 independent experiments.

(D) Gating strategy for murine Plasma cells in the bone marrow. Cells were pre-gated on single cells, living cells and lymphocytes. Subsequently, B220^{-} TACI^{+} CD138^{+} cells were defined as plasma cells.

(E) Quantification of plasma cells in the bone marrow of 43-48 week old mice. On the left, the percentage of plasma cells among living cells is shown. The total cell numbers per hind leg are depicted on the right. Every point represents one mouse, the bars represent the mean values and data are generated from 9-11 mice per genotype within 9 independent experiments.

(F) Quantification of plasma cells of 60 week old mice. On the left, the percentage of plasma cells among living cells is shown. The total cell numbers per hind leg are depicted on the right. Every point represents one mouse, the bars represent the mean values and data are generated from 7-15 mice per genotype within 7 independent experiments.

For statistical analysis, ANOVA test with Šidák post-hoc test was used in case of normal distribution and for non-normally distributed samples, the Kruskal-Wallis test with Dunn's post-hoc test was performed. $p<0.05$(*), $p<0.001$ (**), $p<0.0001$ (***).

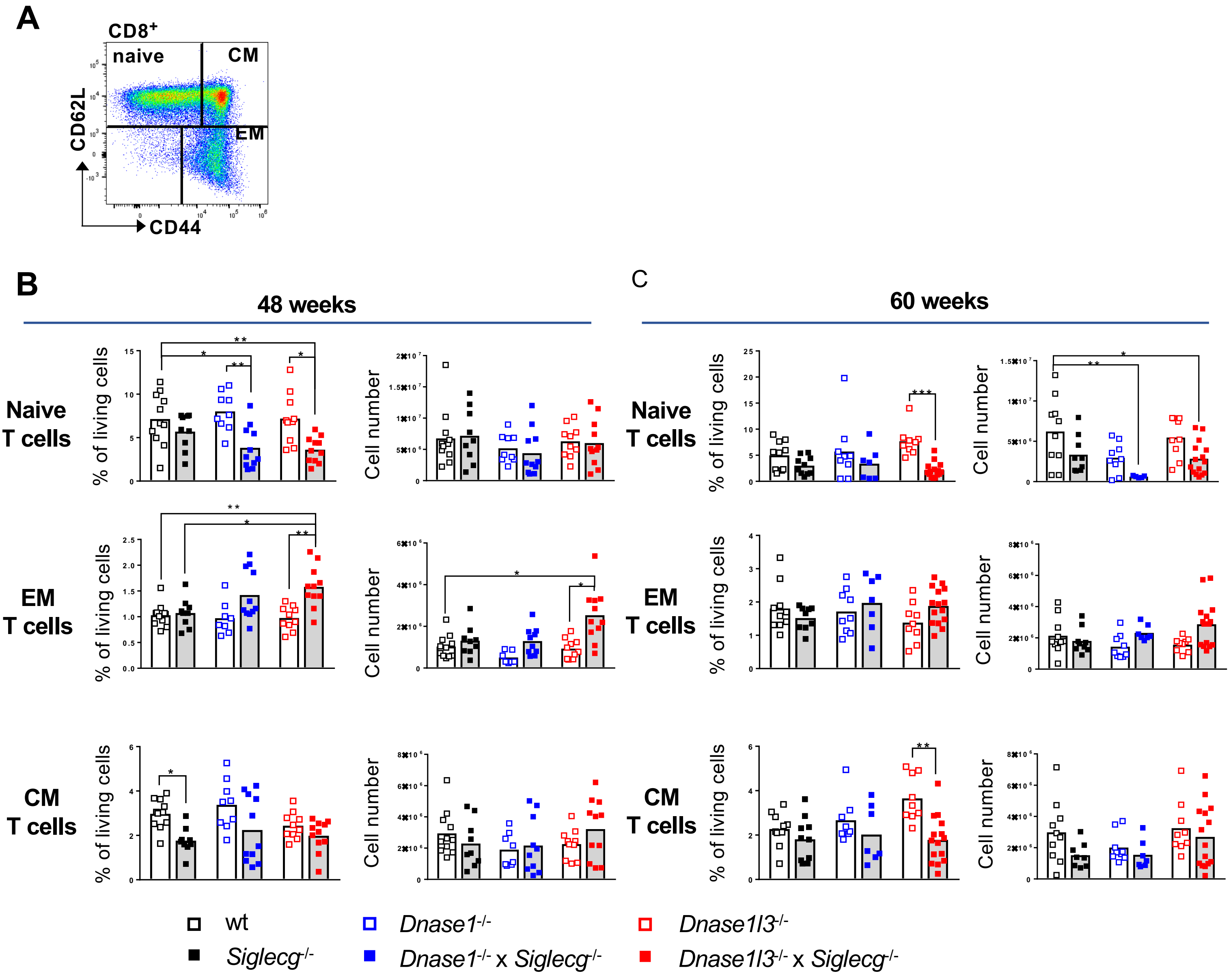

Supplementary Figure 4:

Murine splenocytes were stained with antibodies for $CD8^+$ naïve and memory T cells to analyze them via flow cytometry analysis. Cells of the following genotypes were investigated: WT (black), *Siglecg*$^{-/-}$ (black painted), *Dnase1*$^{-/-}$ (blue), *Dnase1*$^{-/-}$ x *Siglecg*$^{-/-}$ (blue painted), *Dnase1l3*$^{-/-}$ (red), *Dnase1l3*$^{-/-}$ x *Siglecg*$^{-/-}$ (red painted).

(A) Gating strategy for murine $CD8^+$ naïve and memory T cell populations in aging mice. Cells were pre-gated on single cells, living cells and lymphocytes. Subsequently, $CD8^+$ T cells were then classified into naïve ($CD62L^+$, $CD44^{low}$), effector memory (EM; $CD62L^{low}$, $CD44^+$) and central memory (CM; $CD62L^+$, $CD44^+$) T cells based on their CD62L and CD44 expression.

(B) Quantification of $CD8^+$ naïve, EM T cells and CM T cells of 43-48 week old mice. In each case, the left graph shows the percentage of naïve, EM T cells or CM T cells among living cells and the right graph shows the total cell numbers. Every point represents one mouse, the bars represent the mean values and data are generated from 7-11 mice per genotype within 7 independent experiments.

(C) Quantification of $CD8^+$ naïve, EM T cells and CM T cells of 60 week old mice. In each case, the left graph shows the percentage of naïve, EM T cells or CM T cells from living cells and the right graph shows the total cell numbers. Every point represents one mouse, the bars represent the mean values and data are generated from 7-15 mice per genotype within 7 independent experiments.

For statistical analysis, ANOVA test with Šidák post-hoc test was used in case of normal distribution and for non-normally distributed samples, the Kruskal-Wallis test with Dunn's post-hoc test was performed. $p<0.05$(*), $p<0.001$ (**), $p<0.0001$ (***).
